# Evidence for a cryptic parasitoid species reveals its suitability as a biological control agent

**DOI:** 10.1038/s41598-020-76180-5

**Published:** 2020-11-05

**Authors:** M. Lukas Seehausen, Nicolas Ris, Laetitia Driss, Alessandro Racca, Pierre Girod, Sylvie Warot, Nicolas Borowiec, Ivo Toševski, Marc Kenis

**Affiliations:** 1 CABI, rue des grillons 1, 2800 Delémont, Switzerland; 2 Institut Sophia Agrobiotech, INRAE PACA, 400 route des chappes BP 167, 06903 Sophia Antipolis Cedex, France; 3 Present Address: Department of Entomology, Rutgers University, New Brunswick, NJ 08901-8525 USA; 4 Department of Plant Pests, Institute for Plant Protection and Environment, Banatska 33, Zemun, 11080 Serbia

**Keywords:** Ecology, Genetics, Ecology

## Abstract

Uncertainty about the taxonomic status and the specificity of a species commonly prevent its consideration as a candidate for biological control of pest organisms. Here we use a combination of molecular analysis and crossing experiments to gather evidence that the parasitoid wasp *Ganaspis brasiliensis*, a candidate for biological control of the invasive spotted wing drosophila *Drosophila suzukii*, is a complex of at least two cryptic species. Complementary experiments demonstrate that individuals from one genetic group readily parasitize several drosophila species regardless of their food source while individuals from the other one are almost exclusively specific to larvae feeding in ripening fruits. Because only *D. suzukii* attacks ripening fruits in its area of invasion, parasitoids from this second group appear to be well suited as a biological control agent. Our study demonstrates the need for a combination of biosystematics with biological and ecological investigations for the development of safe and efficient biological control programs.

## Introduction

Biosystematic studies play a key role in applied biology, particularly for the safety and efficacy of biological control programs, i.e. the use of natural enemies (predators, parasitoids and pathogens) to reduce pest populations^1,2^. In classical biological control involving the importation of natural enemies from the native area of an invasive species, both the identification of the targeted pest and the biocontrol agent (i.e. natural enemy) are equally important. The targeted pest needs indeed to be clearly identified to be able to search for associated natural enemies in its area of origin. The taxonomic identification of the candidate biocontrol agent is also important to only import the organism with the desired behavioral, ecological, and physiological characteristics, especially to avoid non-target effects. This is a complex task insofar as some of these characteristics (e.g. climatic suitability or host species specificity) can vary between closely related species or even within one species^3–6^. While in some cases the intra-specific differences in such characteristics reflect phenotypic plasticity of a species, in other cases, it is a result of differences between cryptic species, i.e. reproductively isolated populations that have not been reliably identified by morphological characters^7^. For biological control, the latter case is more desirable because the characteristics should be genetically fixed. With the rise in popularity of genetic taxonomic tools, it became clear that cryptic species are more common than previously thought^8^. Thus, the potential to identify safe and efficient biological control agents for the control of invasive species has considerably improved through the use of integrative taxonomy^9,10^.

The spotted wing drosophila (SWD) *Drosophila suzukii* (Matsumura) (Diptera: Drosophilidae) is a frugivorous insect native to Eastern Asia that was accidentally introduced to the Americas and Europe in the 2000s, where it rapidly spread^11^. Unlike sympatric *Drosophila* species in invaded areas, *D. suzukii* females are able to lay eggs inside unwounded ripening fruits due to their sclerotized serrated ovipositor, providing it with a unique niche virtually free from competition^12^. Other biological factors that facilitated the invasion are its broad host range that includes many crops^13^ and non-crop fruits^14^ as well as the absence of coevolved natural enemies able to control the fly in its invasive range^15^. The resulting high abundance of *D. suzukii* is leading to extensive damage, making it a major problem for fruit growers, especially in the soft fruit industry^16^.

Many control strategies, including chemical, cultural, mechanical, and biological control, have been suggested, investigated and applied with various levels of success^11,15,17^. However, losses remain substantial and an area-wide and long-term control strategy is needed^17^. Classical biological control has been proposed as a promising option for control of *D. suzukii*^18,19^, in particular in unmanaged areas, such as wild habitats, public or private gardens, which act as reservoirs^15,17^ due to the current lack of local control methods.

In recent years, several research groups carried out surveys in Asia to find potential biological control agents against *D. suzukii*^18–23^. On the basis of these surveys, several species were identified as attacking *D. suzukii* in Asia, the most abundant ones belonging in *Ganaspis* (Hymenoptera: Figitidae). The morphological examination of hundreds of specimens and comparison with type specimens within this genus led to the affiliation of these Asian strains to *Ganaspis brasiliensis* (Ihering)^24^. Meanwhile, molecular studies revealed a genetic differentiation of the Asian *G. brasiliensis* strains, dividing the species into four to five genetic groups (henceforth called G1–G5), this molecular structuration being comforted by different host specificities^22^, as well as by other differences in biology and behavior of populations from different origins in Asia^25–29^. Using matrix-assisted laser-desorption and ionization time-of-flight mass spectroscopy (MALDI-TOF MS), significant differences in acid-soluble insect protein spectra from four Asian *G. brasiliensis* populations were also found, dividing them into two distinct groups^30^. Altogether, these findings suggest that the taxonomic status of *G. brasiliensis* remains unclear with the existence of regional biotypes or even cryptic species. We therefore adopt here the term “*Ganaspis* cf. *brasiliensis*” to consider this taxonomic uncertainty, as proposed by Girod et al.^19^.

Here, we use DNA barcoding and crossing experiments to determine the existence of cryptic species within *G.* cf. *brasiliensis*. Based on these results, we study the specificity of some *G.* cf. *brasiliensis* populations to *D. suzukii* using no-choice (i.e. one potential host species at a time) and choice tests (i.e. giving the choice between several potential host species). The results are then discussed to assess the suitability of *G*. cf. *brasiliensis* for the use as a classical biological control agent and the applicability of this approach for biological control in general.

## Results

### Molecular characterization

To investigate the molecular clustering within the *G. brasiliensis* complex and to compare it with the genetic groups described by Nomano et al.^22^, two molecular markers were used, the mitochondrial coding gene Cytochrome Oxidase subunit 1 (*COI*) and the nuclear region Internal Transcripted Spacer 2 (*ITS2*). From our samples from China (nine localities samples from five different provinces) and Japan (three localities from different prefectures), we obtained 245 high-quality *COI* sequences distributed in 36 haplotypes, each of which were observed at least twice (see Table S1 and Table S2). Contextualized with relevant GenBank accessions, we obtained an alignment of 62 haplotypes/sequences that were compared for 519 bp.

**Figure 1:**
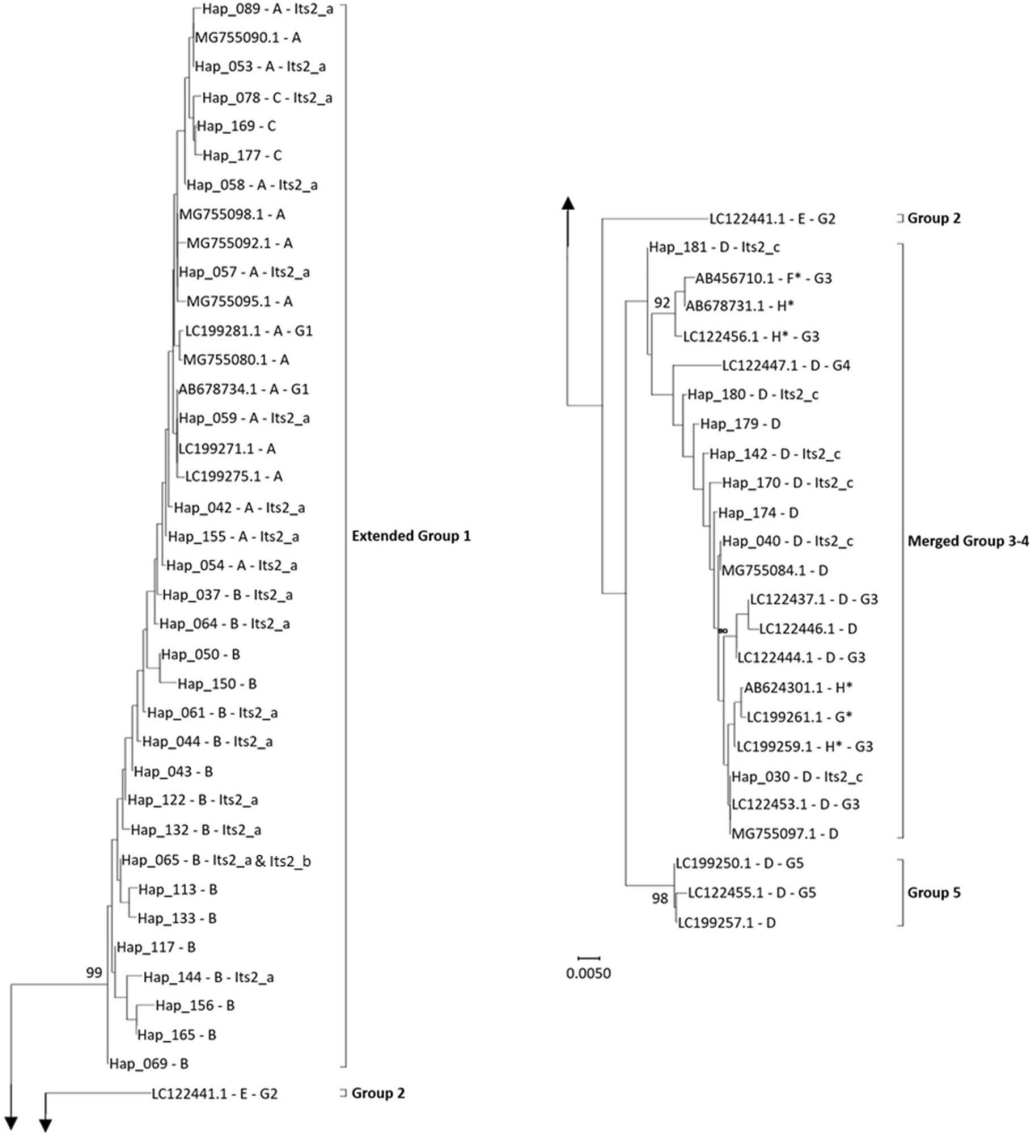
Neighbor-Joining tree for *COI* and correspondence with *ITS2* The labels for haplotypes concatenate information separated by hyphens as follows (from left to right): Either the GenBank accession or a haplotype number referring to internal identifiers in the Institute Sophia Antipolis (see table S2); the related amino-acid haplotype (from A to G) after translation using the invertebrate mitochondrial code; and when available, the related *ITS2* haplotype (its2_a, its2_b, or its2_c) (also see table S2). The haplotypes marked with an asterisk (*) present mutations associated to a change of the biochemical nature of the corresponding amino acid.

At the amino-acid level (i.e. after translation using the genetic code for the mitochondrial of invertebrates), the sequences revealed variations at only five variable codons, generating eight different haplotypes (called A to H in Fig. 1). We suspect the haplotypes F, G, and H of being artefacts or pseudogenes because of the change in the biochemical nature of the substituted amino acid (a polar Threonine instead of a hydrophobic Alanine).

At the nucleotidic level, the three complementary approaches—the Neighbour-Joining tree (Fig. 1), the Maximum Likelihood approach (Fig. S1), and the haplotype network (Fig. S2)—exhibit similar patterns and the clustering is consistent with those previously observed by Nomano et al.^22^. In particular, a highly supported dichotomy distinguishes, on one side, the large molecular cluster hereafter referred to as “extended G1” and a second sub-divided cluster. The extended G1 group included all sequences from Nomano’s group 1 (G1), some sequences from Giorgini et al*.*^23^ originating from the Yunnan province (China), as well as haplotypes recovered by ourselves in several Chinese provinces (Beijing, Guangdong, Shanxi, Sichuan, and Yunnan) and two distant locations in Japan (vicinity of Tokyo and Nara). Within the second cluster, a sub-structuring is observed. In particular, sequences from Nomano’s G2 (geographically restricted to the Iriomote Island, South-West of Japan) and G5 (apparently quite widespread in Japan) appeared well differentiated from others. Finally, a second large cluster (hereafter referred to as “merged G3-4”) was observed including (1) representatives of Nomano’s G3 (various Japanese locations) and G4 (only found in Indonesia), (2) some Chinese (Yunnan) sequences from Giorgini et al.^23^ and (3) some of our own haplotypes found in China (Yunnan only) and Japan (vicinity of Tokyo and Nara). Within this cluster, six accessions (AB456710.1, AB624301.1, AB678731, LC199259.1, LC199261.1 and LC122456.1) appear somewhat doubtful insofar as they are associated to a change in the amino acid sequences (see Fig. 1). Consistently, these sequences are quite isolated in the Maximum Likelihood tree (Fig. S1) and the haplotype network (Fig. S2). Finally, according to the approach used (Neighbour joining, Maximum Likelihood, or haplotype network), the Hap_181 (only found in the Japanese locality Hasuike) appears to be more or less distant from other haplotypes/sequences of the merged Group 3–4 (Fig. 1, Fig. S1, and Fig. S2). However, this haplotype shares the same amino-acid sequence of all other valid haplotypes/sequences in this merged G3-4, supporting its affiliation to this group.

A representative subset of 55 individuals was also characterized for *ITS2* and only three haplotypes were observed on a total of 356–357 bp (Fig. 1). The haplotype its2_a was observed for 43 individuals and was associated to 17 *COI* haplotypes. It was strictly identical to six GenBank accessions including three (AB678771.1, AB678773.1, and LC122343.1) that were affiliated to Nomano’s G1^22^. Haplotype its2_b was observed only once from only a one-way sequencing (failure using the F primer), was identical to its2_a except for one position, and was associated to the *COI* haplotype Hap_065, for which the *ITS2* haplotype its2_a was also observed. The possibility of a technical artefact is thus likely. Haplotype its2_c was observed for 11 individuals and was associated to 6 *COI* haplotypes. It was strictly identical to four GenBank accessions including three (AB678763.1, AB678770, and LC122348.1) that were affiliated to Nomano’s G3^22^. With regard to the merged G3-4 group, the basal *COI* haplotype Hap_181 is associated to the same *ITS2* haplotype (its2_c) as five other *COI* haplotypes found in this study (Fig. 1), once again supporting its affiliation to this group. Because Nomano’s G4 was shown to be characterized by a different *ITS2* haplotype^22^, all the individuals from our study presenting the its2_c haplotype should be affiliated to Nomano’s G3.

**Figure 2:**
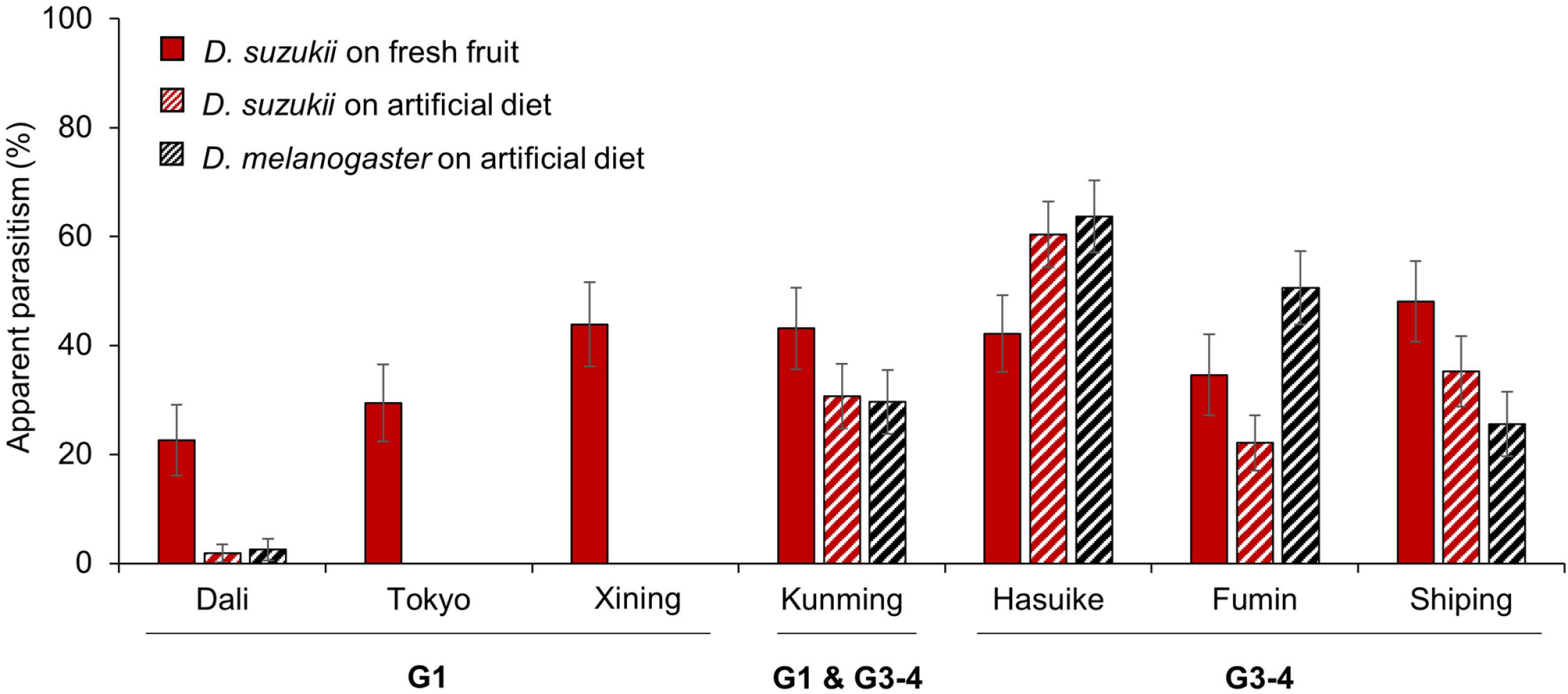
Variation in apparent parasitism by *Ganaspis* cf. *brasiliensis* from different origins in Asia parasitizing two host species feeding on two nutritive media. Three experimental conditions were tested in this no-choice test: *Drosophila suzukii* on blueberry (plain red), *D. suzukii* on artificial diet (striped red), and *D. melanogaster* on artificial diet (striped black). The mean and standard error are indicated for each experimental condition. Parasitoids from different locations in China (Dali, Xining, Kunming, Fumin, and Shiping) and Japan (Tokyo and Hasuike) are grouped according to their molecular affiliation, extended group 1 (G1) and merged groups 3–4 (G3-4), individuals from Kunming being a mix of both groups (see Results and Fig. 1). For each parasitoid origin, 20 replicates per host species-nutritive media combination were tested (total n = 420).

The molecular characterization of individuals from the seven populations that were subsequently used in crossing and behavioral experiments evidenced that all individuals originating from Dali (*COI* haplotypes 054, 058, 078, 169, and 177), Tokyo (059), and Xining (053, 054, 057, and 155) belonged to the extended G1 while those from Fumin (040, 142, 170, 179, and 180), Hasuike (142 and 181), and Shiping (040, 080, and 174) belonged to the merged G3-4. Individuals from Kunming (089 and 143) used in the no-choice experiment (see Fig. 2) have been a mix from these two molecular clusters but those used for all other experiments were found to be from G1 only.

### Crossing experiments

Crossing experiments were done with individuals of two above-described genetic groups with relatively large phylogenetic difference, extended G1 and merged G3-4, to determine if they are able to produce fertile offspring, which would define them as being one species. Based on *CO1*, all tested parental females from Kunming used in this experiment (n = 18) belonged to the extended G1. Individuals from Tokyo and Kunming readily mated and produced female offspring in the first and second generation, irrespective of the female’s origin (Table 1). However, when attempting crosses between G1 from Tokyo and merged G3-4 from Hasuike, mating was never observed and only male offspring was produced by females of either origin. The 0% female offspring was significantly lower for G1 Tokyo × G3-4 Hasuike crosses, when compared to all others (χ^2^ = 281.92; df = 1,7; *P* < 0.0001; Table 1). In control crosses between females and males from the same origin, female offspring was produced, confirming the fertility of the Tokyo and Hasuike populations (Table 1). Thus, the crossing experiment revealed a reproductive isolation between individuals of the two genetic groups.

**Table 1:** Females produced in crossing experiments using *Ganaspis* cf. *brasiliensis* from different genetic groups.

Crossing	Generation	Origin of female	Parental females (n)	Total progeny (n)	Produced females (% ± SE)
G1(T) × G1(K)	F1	G1(T)	9	45	53.3 ± 9.6bc
		G1(K)	19	55	45.5 ± 8.7bc
	F2	G1(T)	24	286	62.6 ± 3.7bc
		G1(K)	20	234	69.2 ± 3.9c
G1(T) × G3-4(H)	F1	G1(T)	18	136	0.0 ± 0.0a
		G3-4(H)	19	159	0.0 ± 0.0a
G1(T) control	F1	G1(T)	15	135	48.1 ± 5.6b
G3-4(H) control	F1	G3-4(H)	12	102	69.6 ± 5.9 bc

Percentage (± standard error) of female progeny produced in the F1 and F2 generation from crossing experiments with *G.* cf. *brasiliensis* individuals of two genetic groups, extended group 1 (G1) and merged group G3-4 (G3-4; after Nomano et al.^20^) and three locations in Asia: (T) Tokyo, Japan; (K) Kunming, China; and (H) Hasuike, Japan. In crosses between females and males of the same origin (controls), only the production of F1 progeny was examined. Percentages with the same lower-case letters are not significantly different at α = 0.05 according to Tukey’s HSD test.

### Affinity towards the targeted host and its nutritive media

To determine if *G*. cf. *brasiliensis* from seven different Asian origins and the two above-described genetic groups (G1 and G3-4) differ in their affinity towards the targeted host and its nutritive media, we conducted a no-choice test, in which each parasitoid female was exposed to one host species—nutritive medium combination at a time. The result showed that an interaction of parasitoid origin and the host’s nutritive medium significantly influenced apparent parasitism (Table S3). Females from the three G1 populations (Dali, Tokyo, and Xining) readily parasitized *D. suzukii* feeding on fresh fruits but only one female parasitized *D. suzukii* and one the non-target host *Drosophila melanogaster* Meigen feeding on artificial diet. Females from the G3-4 populations (Hasuike, Fumin, and Shiping), as well as the mixed population from Kunming, commonly parasitized *Drosophila* feeding on both media (Fig. 2). The mean (± SE) probability of parasitoid females (POF) parasitizing *D. melanogaster* feeding on artificial diet was significantly lower for G1-individuals (2.1 ± 2.1%; n = 47) when compared to G3-individuals (72.2 ± 6.1%; n = 54; χ^2^ = 62.13; df = 1, 99; *P* < 0.0001).

### Influence of the nutritive media on the parasitism of non-target species

**Figure 3:**
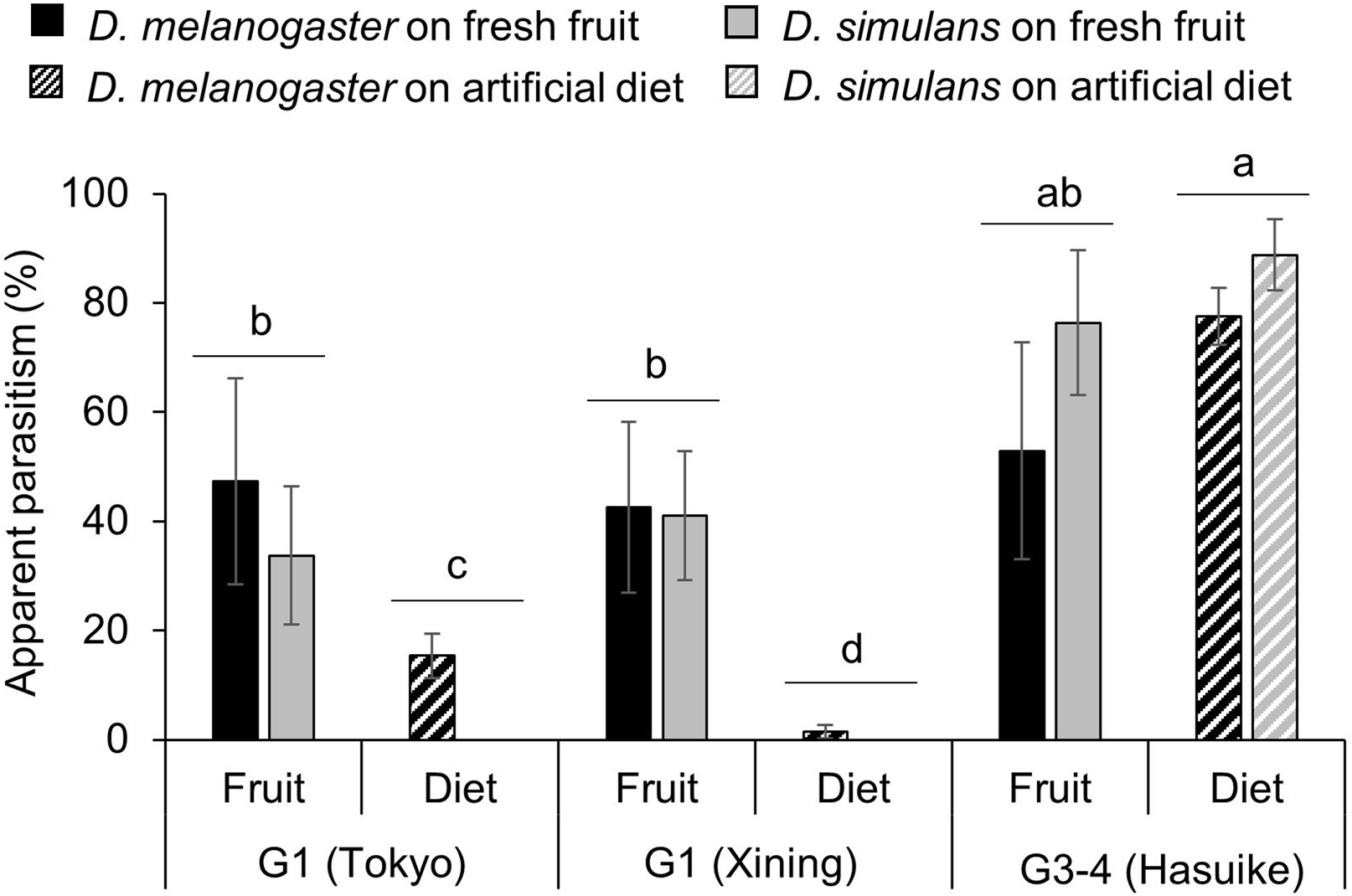
Variation in apparent parasitism by *Ganaspis* cf. *brasiliensis* from two molecular groups parasitizing two non-target host species feeding on two nutritive media. Four experimental conditions were tested in this no-choice test: *Drosophila melanogaster* on blueberry (plain black), *D. simulans* on blueberry (plain grey), *D. melanogaster* on artificial diet (striped black), and *D. simulans* on artificial diet (striped grey). Each condition was tested with parasitoids from different locations in Japan (Tokyo and Hasuike) and China (Xining) comparing two molecular affiliations; extended group 1 (G1) and merged groups 3–4 (G3-4); see Results and Fig. 1. The mean and standard error are indicated for each experimental condition. Bars with the same lower-case letters are not significantly different at α = 0.05 according to Tukey’s HSD test. For each parasitoid origin, 10 replicates per host species-nutritive media combination were tested (total n = 120).

A second no-choice test was conducted including two non-target species, *D. melanogaster* and *Drosophila simulans* Sturtevant, on either artificial diet or blueberries to assess if there are differences between the two genetic groups of parasitoids in regard of parasitism of non-target species on different nutritive media. The experiment revealed a significant interaction between parasitoid origin and host species, as well as between origin and nutritive medium (Table S4). For the two G1 populations (Tokyo and Xining), parasitism of both host species was significantly higher on fruits when compared to artificial diet, while for the G3-4 population from Hasuike, there was no significant difference in parasitism of both host species between fruit and artificial diet (Fig. 3).

### Preference for the targeted host and its habitats

**Figure 4:**
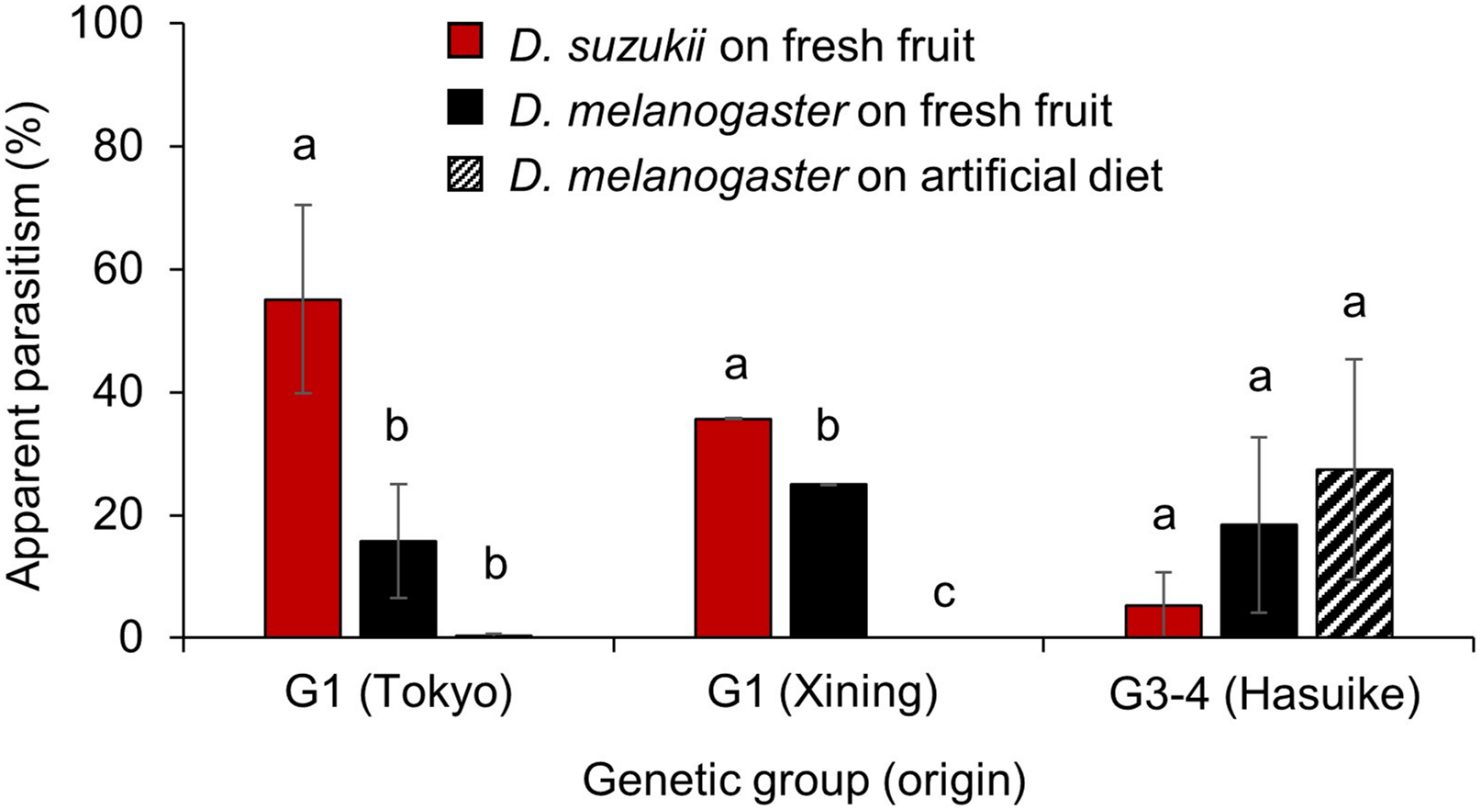
Variation in apparent parasitism by *Ganaspis* cf. *brasiliensis* from two molecular groups parasitizing two host species and two nutritive media in three-choice tests. Three experimental conditions were tested in this choice test: *Drosophila suzukii* on blueberry (plain red), *D. melanogaster* on blueberry (plain black), and *D. melanogaster* on artificial diet (striped black). The tests were done with parasitoids from two genetic groups, extended group 1 (G1) and merged groups 3–4 (G3-4; see Results and Fig. 1) originating from different locations in Japan (Tokyo and Hasuike) and China (Xining). The mean and standard error are indicated for each experimental condition. Bars with the same lower-case letters are not significantly different at α = 0.05 according to Tukey’s HSD test. In total, 68 female wasps were tested 20 originating from Hasuike, 24 from Tokyo, and 24 from Xining.

To determine if the difference between the two genetic groups of parasitoids in their affinity to the nutritive medium of the host also holds true when given the choice between different host species and media, a three-choice bioassay was conducted, with the choices being *D. suzukii* on blueberry, *D. melanogaster* on blueberry, and *D. melanogaster* on artificial diet. The results from the bioassay showed a significant difference in parasitism between choices for the G1 populations (Tokyo and Xining), but not for the G3-4 population (Hasuike; Table S5). For both G1 populations, parasitism of *D. suzukii* on fruits was significantly higher compared to *D. melanogaster* on fruits or artificial diet. Only one G1 female from Tokyo and none from Xining attacked *D. melanogaster* on artificial diet (Fig. 4).

**Figure 5:**
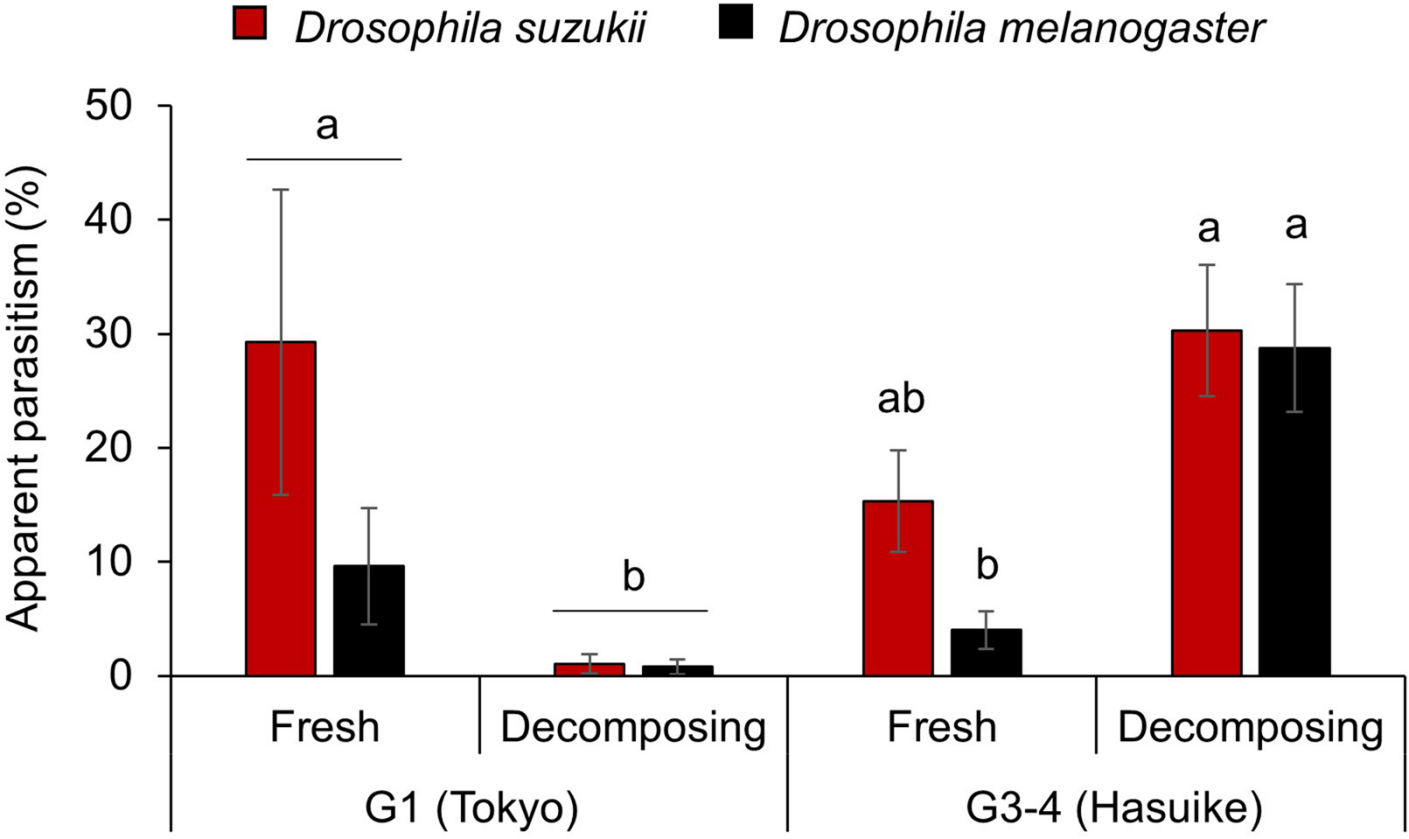
Variation in apparent parasitism by *Ganaspis* cf. *brasiliensis* from two molecular groups parasitizing two host species on fresh and decomposing fruits in four-choice tests. Four experimental conditions were tested in the choice tests: all combinations of the hosts *Drosophila suzukii* (red) and *D. melanogaster* (black) feeding on fresh and decomposing blueberries. The tests were done with parasitoids from two genetic groups, extended group 1 (G1) and merged groups 3–4 (G3-4; see Results and Fig. 1), originating from two locations in Japan (Tokyo and Hasuike). The mean and standard error are indicated for each experimental condition. Bars with the same lower-case letters are not significantly different at α = 0.05 according to Tukey’s HSD test. In total, 27 and 22 females originating from Tokyo and Hasuike were tested, respectively.

Additionally, a four-choice bioassay was conducted to assess if the habitat specificity of G1 and the generality of G3-4 *G.* cf. *brasiliensis* also hold true when offering the more natural choices of fresh and decomposing fruits. In this bioassay, opposite responses in parasitism between the genetic groups G1 and G3-4 were recorded: G1 from Tokyo parasitized hosts in fresh fruits significantly more often than in decomposing fruits, irrespective of the tested host species (*D. suzukii* and *D. melanogaster*; Table S6). For G3-4 from Hasuike, however, there was a significant interaction between host species and status of the fruit (Table S6), with a significantly higher parasitism of *D. melanogaster* feeding on decomposing fruits, when compared to fresh fruits (Fig. 5).

## Discussion

We present strong evidence that the parasitoid described as *G. brasiliensis*^24^ is a species complex, which is in accordance with findings from other recent studies^22,30^. The evidence leading to this statement relies on three criteria: molecular divergence, reproductive incompatibilities, and differences in ecological characteristics.

At the molecular level, three main results were obtained from our joint analysis of *COI* sequences/haplotypes from three independent sources^22,23^ (and this study). Firstly, we confirmed at least four of the five groups defined by Nomano and colleagues^22^, the sole difference being the clustering of Nomano’s G4 within G3. This is however likely due to a lack of power in our phylogenetic reconstruction insofar as, within our merged G3-4, sequences from the Nomano’s G4 differ from all others on *ITS2*. Moreover, it is worth noting that only few information is available about Nomano’s G4, from which rarely available specimens were only observed in Indonesia. Secondly, the addition of new individuals from already sampled but also new Chinese and Japanese areas allowed to have access to a greater molecular diversity within the two main clusters, the extended G1 and the merged G3-4. Despite this, the molecular divergence observed using the Kimura 2 parameters distance between the two clusters remains strongly supported with a mean level of divergence of 5.6% (Table S7). By comparison, the mean within-cluster divergences are only 1.0% (max: 2.7%) and 0.9% (max: 2.5%) for the extended G1 and the extended G3 (i.e. once Nomano’s group 4 and the doubtful sequences are discarded), respectively. Thirdly, our study speaks in favor of a certain prudence with regard to the use of *COI* sequences in *Ganaspis* species. Several authors have indeed pointed out the risks of pollution by “*COI*-like sequences”^31^. Based on our own work and sequences already deposited in GenBank, we highly suspect that the *Ganaspis* genus is sensitive to this issue. Comparatively, *ITS2* or multi-loci studies may be more reliable for this taxon.

At the reproductive level, our study evidenced a complete reproductive isolation between G1 individuals originating from Tokyo, Japan, and G3-4 individuals from Hasuike, Japan. Based on our observations, this reproductive incompatibility seems to be caused by a pre-mating isolation. By contrast, G1 individuals from Tokyo were fully compatible with Chinese representatives of the same molecular cluster. These results thus complement those obtained by Nomano et al.^22^, who evidenced a strong although not total isolation between one G3 and two G5 representatives. The two reproductively isolated genetic groups have been shown to occur sympatrically at least in Tokyo^22^, Nara (this study), and the Yunnan province of China^23^. We show here that sympatry even occurs at the same sites, as shown by the mixed populations from Kunming in China and data from Giorgini et al.^23^. The fact that two genetically distinctive groups occur sympatrically lends further evidence to the existence of cryptic species, although morphological differences have yet to be found.

At the ecological level, females from the two genetic groups displayed differences in host species and habitat specificity. Originally, G1 *G*. cf. *brasiliensis* were described as specific to *D. suzukii*, and G3 females as more generalist^22,23,32^. Our no-choice test with *G*. cf. *brasiliensis* from different origins showed that G1 females rarely parasitize *D. melanogaster* feeding on artificial diet but G3-4 females readily do. This result corroborates that the possible existence of cryptic species explains the previously found differences in specificity between host larvae feeding on fresh fruit and artificial diet^25,26^. However, our results from no-choice and choice experiments show that this difference is a habitat specificity to fruits by G1 females, rather than a host specificity, as the physiological host range (i.e., all host species that support development and sustain feeding by the parasitoid) of *G*. cf. *brasiliensis* from both genetic groups includes *Drosophila* species other than *D. suzukii*. Nevertheless, for *Drosophila* larvae feeding on fruits, G1 females prefer to parasitize *D. suzukii*, when compared to *D. melanogaster*. G3-4 females on the other hand, parasitized all species-nutritive medium combinations to the same extent. Moreover, when comparing parasitism of target and non-target species feeding on either fresh or decomposing fruits, the results showed that while G1 females readily parasitized both *Drosophila* species feeding on fresh fruits, parasitism of the same hosts feeding on decomposing fruits was rare. In contrast, there was no difference in parasitism of *D. suzukii* between the two nutritive media for G3-4 females and *D. melanogaster* was parasitized more often on decomposing fruits when compared to fresh ones. During several surveys in Asia, *G.* cf. *brasiliensis* were always only collected from fresh ripe fruits and were never obtained from fruit baits, such as sliced sections of banana^18,19,23,33^, indicating a high degree of habitat specificity to fresh fruits under field conditions.

Our findings have profound implications for the use of *G*. cf. *brasiliensis* as a biological control agent in *D. suzukii*’s area of invasion. Due to the parasitoid’s high abundance and importance as a mortality factor of *D. suzukii* in Asia^18,19,23^, as well as under laboratory conditions^25–28^, this wasp has been repeatedly suggested as a biological control agent against *D. suzukii*. However, uncertainties concerning its taxonomic status and degree of specificity have so far cast doubt on its suitability for classical biological control. *Drosophila* species play important ecological roles as decomposers and as part of complex trophic networks^34^. Heavy parasitism of these native fruit flies by an imported generalist biological control agent might have negative consequences on the ecosystem. Therefore, uncertainties about the specificity of a classical biological control agent can be seen as a risk for undesired non-target effects. The discovery that *G*. cf. *brasiliensis* might be a species complex of at least two congeneric species and that one of them can clearly be associated with a higher habitat specificity to fresh fruits, shows the suitability of G1 *G*. cf. *brasiliensis* to control *D. suzukii* and allows to focus on that group for efforts to develop a classical biological control program. *Drosophila suzukii* is known to oviposit and develop in both fresh and decomposing fruits but has its rather unique niche in fresh fruits^35^. Its broad host range allows it to attack fresh fruits throughout the year^14,36^, thus, it does not need to move from fresh to decomposing fruits during the season. The difference in natural habitat between immature *D. suzukii* developing in fresh ripe fruits and other frugivorous *Drosophila* species in decomposing fruits^12^ might translate the habitat specificity of G1 *G*. cf. *brasiliensis* into a host specificity. If entirely specific to ripe fruits under natural conditions, G1 wasps will only parasitize *D. suzukii* in its invasive range. To confirm this hypothesis, subsequent studies should further investigate the specificity of G1 *G.* cf. *brasiliensis* to hosts in fresh fruits under natural conditions in Asia. This might also be a chance to do complimentary genetic studies with field-collected material as described by Condon et al.^37^ to gather further evidence for the existence of cryptic species within *G.* cf. *brasiliensis*. Additionally, the distribution of G1 *G.* cf. *brasiliensis* in Asia should be assessed accurately to find out if this parasitoid is well adapted to temperate climates and can therefore successfully establish as a biological control agent against *D. suzukii* in temperate regions of Europe and the Americas. More studies are also needed about the phenology of G1 *G*. cf. *brasiliensis* and its synchrony with the target pest in the invaded range.

The use of integrative characterization, i.e. the combination of genetic taxonomic tools with biological and ecological studies^9,10^, is a useful approach to investigate inter- and intra-specific differences of key characteristics of natural enemies that are studied as potential biological control agents^3,4,6^. Several other case studies highlight the general need for this approach in biological control. Using genetic identification approaches, it has been suggested that multiple generalist parasitoids of the subfamily Aphidiinae (Hymenoptera: Braconidae) are in fact composed of cryptic species, each associated with a different host species^5^. Together with crossing experiments and more thorough studies of the parasitoids’ biology, these results may help to identify specific biological control agents against aphids that otherwise would have been disregarded because of taxonomic uncertainties. Integrative taxonomy and phylogeny play also a crucial role in matching natural enemies with their hosts for successful classical biological control. A great example is the use of this approach to find a classical biological control agent in Southern Africa against a mealybug that is invasive in Spain^38^. Another example is the thorough molecular analysis which revealed that the invasive eucalyptus weevil that was identified as *Gonipterus scutellatus* (Coleoptera: Curculionidae) is composed of a complex of sister species with different distributions in their area of origin, Australia^39^. Because the biological control agent *Anaphes nitens* (Hymenoptera: Mymaridae) was originally collected from a *Gonipterus* sp. occurring in the relatively warm Southern Australia, it is ecologically mismatched with *Gonipterus* spp. weevils in colder climates of some release countries, leading to inconsistent success in controlling the weevils^40^. Finally, in search for parasitoids as suitable biological control agents against *Pissodes* spp. (Coleoptera: Curculionidae) weevils, several sibling species of the genus *Eubazus* (Hymenoptera: Braconidae) have been discovered, some of which are specific to weevils in exclusive microhabitats^41^. However, within these sibling species, differences in diapause induction exist, synchronizing only one biotype of the parasitoid with the target host, making it the most suitable candidate for biological control^42^. Together with our results, these examples highlight the importance of integrated biosystematic studies to increase the efficacy in finding suitable candidates for classical biological control against invasive pests.

## Methods

### Drosophila rearing

The starting colony of *D. suzukii* was collected from wild *Rubus* sp. and *Fragaria* sp. fruits in various sites in Switzerland in 2015^23^. The flies from the initial collection are described molecularly by Fraimout et al.^43^. The starting colony of *D. melanogaster* and *D. simulans* were obtained from laboratory colonies of INRA (Sophia-Antipolis, France) in 2015 and 2019, respectively. The general rearing of flies was done in plastic tubes (5 cm diameter, 10 cm height) containing approximately 10 g of artificial diet (Formula 4-24 medium, Carolina Biological SupplyCo., Burlington, NC), 40 ml of methyl-4-hydroxylbenzoate solution (1.43 g/L) to inhibit fungal growth, and a few grains of commercial instant dry yeast. The tubes were kept in growth chambers at 22 ± 2 °C, 60% ± 10% RH, and a 16 h photoperiod (hereafter called general rearing conditions). To collect eggs and resulting larvae on different nutritive media (i.e., fresh and decomposing fruits or artificial diet) for the below-described parasitoid rearing and experiments with parasitoids, some adult flies were kept in gauze cages (BugDorm-4F4545) at general rearing conditions. They were fed with sugar water provided on dental cotton rolls and dried instant yeast, additional water was provided on cellulose paper. The nutritive media were exposed to adult flies when needed.

### Parasitoid rearing

The starting colonies of *G*. cf. *brasiliensis* were obtained during surveys in Asia from 2015–2017 and names to describe their origin are based on the collection sites described by Girod et al.^19^: Dali, Fumin, Kunming, Shiping, and Kunming—Xining temple (Xining in this study) in the Yunnan Province of China, as well as Hasuike (Nagano) and Tokyo—Naganuma park (actually on the territory of Hachioji but named Tokyo in this study) in Japan. The parasitoids were reared in the quarantine laboratory at CABI-Switzerland (Delémont, Switzerland) separated by origin in gauze cages (BugDorm-4F4545) to prevent them from interbreeding. The general rearing was done on *D. suzukii* larvae feeding on blueberries as described by Girod et al.^19^, with the difference that fruits were only exposed for 24 h to *D. suzukii* for oviposition. The environmental parameters of the quarantine chamber were the above-described general rearing conditions. Up to 50 adult wasps were kept in transparent plastic containers (9 cm diameter, 5 cm height) inside each gauze cage. An Eppendorf tube with a wet cellulose paper was added as a water source and the container was closed with a foam plug on which a drop of honey was placed as food source. Six fresh blueberries, which were placed 24 h before in the *D. suzukii* rearing cages to collect eggs, were added every 2–3 days to each container with adults to allow for parasitism of young fly larvae. After the exposure to the wasps, infested fruits were removed from the containers and kept in clear plastic tubes (5 cm diameter, 10 cm height) with a filter paper at the bottom to absorb leaking fruit juice. Every 2–3 days, the presence of newly hatched wasps was checked among rearing tubes and adult wasps were transferred to the oviposition containers.

### Molecular characterization

The molecular characterization was performed on (1) individuals originating from the field (nine locations from five provinces in China and three locations from three prefectures of the Honshu island in Japan), (2) the derived laboratory strains and (3) individuals used for the experiments (Table S2). Two molecular markers were used, the mitochondrial coding gene Cytochrome Oxidase subunit 1 (*COI*) and the nuclear region Internal Transcripted Spacer 2 (*ITS2*). Both were previously used to characterize *Ganaspis* individuals from Eastern Asia^22,23^ and elsewhere.

The DNA was extracted in a total of 30 µl using either the prepGEM Insect kit (Zygem) (3 h at 75 °C and 5 min at 95 °C), or the QuickExtract DNA Extraction Solution (n°QE09050, Lucigen) (15 min at 65 °C and 2 min at 98 °C). For both molecular markers (*COI* and *ITS2*), each individual PCR was realized in a total of 25 µl, including 12.5 µl of the Multiplex PCR Master Mix (Qiagen), 0.125 µl of each primer (100 µM), and 1 µl DNA. For *COI*, the primers LCO (5′-GGTCAACAAATCATAAAGATATTGG-3′) and HCO (5′-TAAACTTCAGGGTGACCAAAAAATCA-3′)^44^ were used for more than 400 individuals. PCR conditions consisted of (1) 15 min at 95 °C, (2) 35 cycles of 30 s at 94 °C, 90 s at 50 °C and 60 s at 72 °C, (3) 10 min at 72 °C. For ITS2, the primers ITS2-F (5′-TGTGAACTGCAGGACACATG-3′) and ITS2-R (5′-AATGCTTAAATTTAGGGGTA-3′)^45^ were used for a subset of representative individuals. PCR conditions consisted of (1) 15 min at 95 °C; (2) 40 cycles of 30 s at 94 °C, 90 s at 53 °C, and 60 s at 72 °C; and (3) 10 min at 72 °C. In both cases, the PCR was checked using a QIAxcel DNA Fast Analysis Kit on a QIAxcel Advanced System (Qiagen). Positive PCR products were then sequenced with the Sanger method in one direction with the HCO primer for *COI* and both directions for *ITS2*. Sequences were trimmed, assembled and aligned using ClustalW for *COI* and Muscle for *ITS2* (Geneious, version 10.2.3). For *COI*, only haplotypes observed twice within the panel of high-quality sequences (length > 520 bp and no undetermined nucleotide) were considered. These data were then enriched with 83 additional GenBank accessions, including in particular sequences from Nomano et al.^22^ and Giorgini et al.^23^. The whole dataset (our own haplotypes and GenBank accessions) was then analyzed on a common part of 519 bp included between the two marks, ATTGGDTCAA and TTAGCAGGTG (5′ → 3′ on the positive strand). Three criteria were then applied to summarize and clean the data including: (1) the conservation of repres entative, necessary and sufficient sequences from the three main sources^22,23^ (and this study); (2) the exclusion of sequence with undetermined nucleotide(s); (3) the exclusion of each sequence with a unique amino-acid sequence. A final dataset of 62 sequences (haplotypes from this study and GenBank accessions) remained after this process. Based on this dataset, three complementary approaches were used to investigate the molecular clustering: (1) a Neighbour Joining approach using the Tamura 3 parameters distance (the best evolutionary model according to the software MEGA10.1.7^46^), using 500 replicates for bootstrapping; (2) a Maximum Likelihood approach using the evolutionary model HKY85 + I (the best model according to the software PhyML3.0^47^); and (3) the constitution of a network using the Median Joining method (ε set to zero, PopArt^48^). The Kimura 2 parameters distance (often used in the frame of barcoding’s studies) was also used to investigate the pairwise distances within and between clusters (see Discussion). For *ITS2*, the identified haplotypes were directly compared to those available on GenBank and mapped into the *COI* Neighbor-Joining tree.

### Crossing experiments

*Ganaspis brasiliensis* is arrhenotokous, unmated females produce only male progeny while mated females are able to produce both males (unfertilized eggs) and females (fertilized eggs). Thus, the proportion of female progeny can be used as an indicator of reproductive isolation. With regard to already acquired knowledge on Asian *Ganaspis* cf. *brasiliensis*^19,22,25,30^, we more precisely investigated here the reproductive (in)compatibilities between the two main molecular clusters (G1 and G3-4—see Results and Discussion) and, within the cluster G1, between two geographically distant populations (one Chinese and one Japanese). Thus, crossing experiments with individuals from three locations were done here: Tokyo, Hasuike and Kunming. For the latter, only individuals that were a posteriori affiliated to G1 through the molecular characterization described above were taken into account. For individuals from each location, parasitized *Drosophila* pupae from the general parasitoid rearing (see above) were identified under a microscope (parasitoid pupae can be seen through the translucent *Drosophila* pupal case) and kept individually in plastic vials containing moisturized plastic foams. Within 24 h after emergence, 1–2 males were placed with each virgin female during 24 h for mating. Females were then transferred to a plastic vial containing 10–30 first instar *D. suzukii* larvae feeding in fresh blueberries and drops of honey for the parasitoid’s nutrition. After 3 days, females were collected and kept in 95% ethanol for potential molecular analysis. The vials containing the potentially parasitized *D. suzukii* larvae in blueberries were kept until adult emergence under the general rearing conditions described above. Upon emergence of the F1 generation, adults were sexed based on antennal length (males have longer antennae than females^24^) and the percentage of female progeny was calculated for each parental female. To test the fertility of F1 females, they were allowed mating with males from the same origin for 24 h. Then, the above described oviposition procedure was repeated, and upon emergence, the F2 progeny was sexed and percentage of females was calculated. The number of parental females for each crossing varied from 9–24 (Table 1), depending on emergence during the experimental period.

### Affinity towards the targeted host and its nutritive media

To study the specificity of *G*. cf. *brasiliensis* from the above mentioned seven different origins in Asia, three combinations of hosts and nutritive media were tested under no-choice conditions: (1) *D. suzukii* larvae feeding on blueberries, (2) *D. suzukii* larvae feeding on artificial diet, and (3) *D. melanogaster* larvae feeding on artificial diet. The blue formula of the above-mentioned artificial diet was used to facilitate counting of *Drosophila* eggs. Additionally, the diet was blended with about 25 g of fresh blueberries, as described by Girod et al.^25^. The artificial diet and fresh blueberries were exposed to the respective *Drosophila* species for 1–3 h, until 10–30 eggs were counted under a microscope, and incubated for 24 h at room temperature to allow eggs to hatch. Mated and naïve (i.e., never exposed to hosts for oviposition) 3–4 d old *G.* cf. *brasiliensis* females were then released individually into plastic tubes (2.7 cm diameter, 5.2 cm height) containing one of the three media. The tubes were closed with a moist foam lid containing a drop of honey to nourish the parasitoids. Females were removed from the tubes after 48 h and placed in 95% ethanol for genetic identification based on *CO1*, as described above. The tubes containing potentially parasitized *Drosophila* larvae were kept at the general rearing conditions and observed for fly and parasitoid emergence on a regular basis for 40 d. For each tube, the number of *Drosophila* flies and parasitoids were recorded. For each parasitoid origin, 20 replicates per host species-nutritive media combination were tested, for a total of 420 individual females.

### Influence of the nutritive media on the parasitism of non-target species

A second no-choice test was done to investigate whether *G*. cf. *brasiliensis*’ host specificity is dependent on the nutritive medium of the host. To this end, four host species-nutritive medium combinations were tested: *D. melanogaster* or *D. simulans* larvae feeding on either blueberries or artificial diet. Because both *Drosophila* species do not have a serrated ovipositor and can therefore not oviposit through the skin of fresh fruits, slightly decomposed blueberries were cut in half and exposed to these species until 10–30 eggs were counted on each half. As in the first no-choice test, the artificial diet used in this experiment was the blue formula blended with about 25 g blueberries. The experiment was then conducted as described above for the first no-choice test, with the difference that 10 replicates for each host species-nutritive medium combination were used for parasitoids originating from Tokyo, Xining, and Hasuike only. This brought the total number of females for this experiment to 120.

### Preference for the targeted host and its habitats

To investigate differences in preferences for the targeted host and its habitats among the different genetic groups of *G*. cf. *brasiliensis*, a three- and a four-choice bioassay were done. The bioassays took place in a cylindrical transparent plastic container (10 cm diameter, 5 cm height) with two holes of 2.5 cm diameter in the lid: one was covered with netting for ventilation and the other closed with a foam plug on which a drop of honey was placed to nourish the parasitoid. Inside each container, one 4–5 days old mated parasitoid female was placed, a plastic vial with wet cellulose paper as a water source, and small dishes (2.5 cm diameter, 1 cm height) containing the choices for oviposition in a random order. To avoid the influence of light and colors on the wasp’s directional choice, the choice arenas were placed inside a white plastic box (100 × 50 cm), leaving only one light source from above. After 24 h in the choice arena at the general rearing conditions, female parasitoids were kept in 95% ethanol to allow for further DNA analysis confirming the genetic group they belonged to. The dishes containing the different hosts and nutritive media were placed separately in rearing tubes (5 cm diameter, 10 cm height) containing a moist filter paper at the bottom and covered with a moist foam lid to avoid drying of the media. Three weeks after the beginning of the choice test, all adult *Drosophila* were removed from the rearing tubes and were counted. Until the eighth week after the choice test, emerging parasitoids were collected once a week, sexed, and counted.

The three-choice bioassay was designed to determine if also when given the choice, G1 *G*. cf. *brasiliensis* are specific to fruits as the host’s nutritive medium, rather than to the host species, while G3-4 parasitoids are not specific to either. Therefore, the three host-species-nutritive medium combinations were (1) *D. suzukii* or (2) *D. melanogaster* larvae feeding on fresh blueberry, and (3) *D. melanogaster* larvae feeding on artificial diet. All media were prepared as described above for the no-choice experiments. In total, 68 female wasps were tested in the three-choice bioassay, 20 originating from Hasuike, 24 from Tokyo, and 24 from Xining.

To determine if the habitat specificity of G1 and generality of G3-4 *G*. cf. *brasiliensis* also hold true when comparing fresh to decomposing fruits, a four-choice bioassay was designed. The host species-nutritive media combinations were (1) *D. suzukii* or (2) *D. melanogaster* larvae feeding on either (3) fresh or (4) decomposing blueberry. Infestation of fresh blueberries with fly larvae was done as described above. To decompose fruits, blueberries were exposed to room temperature in a plastic container for 7–10 days until growth of molt was visible. They were then exposed to *D. suzukii* and *D. melanogaster* for the collection of eggs as described for fresh fruits. In total, 27 and 22 females originating from Tokyo (G1) and Hasuike (G3-4) were tested, respectively, in the four-choice bioassay. For all choice tests, only results from females that produced at least one offspring were analyzed.

### Statistical analysis

Apparent parasitism (AP) was calculated as the proportion of parasitoid offspring among the total number of insects that emerged from the nutritive medium (i.e. *Drosophila* sp. and parasitoids). The proportion of ovipositing females (POF) was calculated as the number of female parasitoids which produced at least one offspring (or which showed an oviposition response, in the case of the behavioral experiments) divided by the number of females tested. All data were analyzed using logistic regression followed by post-hoc comparisons of means with Tukey adjustments. Differences in proportions of females in the crossing experiment as well as AP and POF in the no-choice experiments was analyzed using quasibinomial distributions to account for overdispersion of the residuals (*glm* function of the ‘stats’ package in R^49^). For the no-choice experiment with parasitoids from different origins, AP was analyzed with the explanatory variables parasitoid origin, nutritive medium, and their interaction; and the POF developing on *D. melanogaster* feeding on artificial diet was analyzed with the parasitoid’s genetic group (G1 or G3-4) as explanatory variable. AP in the no-choice experiment with non-target species, the explanatory variables were parasitoid origin, host species, nutritive medium, and all possible interactions.

Mixed effects logistic regressions (*glmer* function of the ‘lme4’ package in R^50^) were used to analyze AP in the choice tests. Analyses were done for each parasitoid origin separately because of convergence problems with more than one fixed effect. Therefore, nutritive medium was the sole fixed-effect explanatory variable for all analyses concerning the choice tests. In all cases, individual females were included as a random effect to account for correlation of parasitism between the media by the same female and an additional observation-level random effect was introduced to solve the problem of residual overdispersion.

## Supplementary information

Supplementary Information
