# Supplementary material for: Evidence for a cryptic parasitoid species reveals its suitability as a biological control agent
Source: Sci Rep. 2020 Nov 5;10:19096. doi: 10.1038/s41598-020-76180-5 (PMC7645786; doi:10.1038/s41598-020-76180-5)
Supplement: Supplementary file 1 — Supplementary Information [file 41598_2020_76180_MOESM1_ESM.docx]

**Supplementary information**

**Evidence for a cryptic parasitoid species reveals its suitability as a biological control agent**

M. Lukas Seehausen^1*^, Nicolas Ris^2^, Laetitia Driss^1^, Alessandro Racca^1^, Pierre Girod^1,3^, Sylvie Warot^2^, Nicolas Borowiec^2^, Ivo Toševski^1,4^, Marc Kenis^1^

^1^CABI, rue des grillons 1, 2800 Delémont, Switzerland

^2^Institut Sophia Agrobiotech, INRAE PACA, 400 route des chappes BP 167, 06903 Sophia Antipolis Cedex, France

^3^Present address: Department of Entomology, Rutgers University, New Brunswick, NJ 08901-8525, United States of America

^4^Department of Plant Pests, Institute for Plant Protection and Environment, Banatska 33, Zemun 11080, Serbia

*Corresponding author: l.seehausen@cabi.org

**Table S1: Cytochrome Oxidase subunit 1 (*COI*) haplotypes of *Ganaspis* cf. *brasiliensis* in sampled Chinese and Japanese locations.** Distribution of amino acid haplotypes found after analysis of the molecular marker *COI* from *G*. cf. *brasiliensis* individuals originating from different Chinese and Japanese locations. “Extended Group 1” and “Extended Group 3” refer to the molecular clustering observed in this study (see Results and Fig. 1). The haplotype groups A-D refer to variations in variable codons.

| **Country** | **Province** | **Location** | **Extended Group 1** | | | | | | |  |  |  |  |  |  |  |  |  |  |  |  |  |  |  |  |  |  |  |  |  |  |  |  | **Extended Group 3** | | | | | | |  |
| --- | --- | --- | --- | --- | --- | --- | --- | --- | --- | --- | --- | --- | --- | --- | --- | --- | --- | --- | --- | --- | --- | --- | --- | --- | --- | --- | --- | --- | --- | --- | --- | --- | --- | --- | --- | --- | --- | --- | --- | --- | --- |
|  |  |  | Haplotype A | | | |  |  |  |  |  | Haplotype B | | | |  |  |  |  |  |  |  |  |  |  |  |  |  |  | Haplotype C | | |  | Haplotype D | | | |  |  |  |  |
|  |  |  | Hap_042 | Hap_053 | Hap_054 | Hap_057 | Hap_058 | Hap_059 | Hap_089 | Hap_155 |  | Hap_037 | Hap_043 | Hap_044 | Hap_050 | Hap_061 | Hap_064 | Hap_065 | Hap_069 | Hap_112 | Hap_113 | Hap_117 | Hap_132 | Hap_133 | Hap_144 | Hap_150 | Hap_156 | Hap_165 |  | Hap_078 | Hap_169 | Hap_177 |  | Hap_040 | Hap_080 | Hap_142 | Hap_170 | Hap_174 | Hap_179 | Hap_180 | Hap_181 |
| **China** | Beijing | Jiu |  |  | 1 |  | 2 |  |  |  |  |  |  |  |  |  |  |  |  |  |  |  |  |  |  |  |  |  |  |  |  |  |  |  |  |  |  |  |  |  |  |
|  | Guangdong | Yangtai |  | 1 |  |  |  |  |  |  |  |  |  |  |  |  |  |  |  |  |  |  |  |  |  |  |  |  |  |  |  |  |  |  |  |  |  |  |  |  |  |
|  | Shanxi | Xining |  | 1 | 1 | 7 |  |  |  | 8 |  |  |  |  |  |  |  |  |  |  |  |  |  |  |  |  |  |  |  |  |  |  |  |  |  |  |  |  |  |  |  |
|  | Sichuan | Dazhou |  | 1 |  | 1 |  |  |  |  |  |  |  |  |  |  |  |  |  |  |  |  |  |  |  |  |  |  |  |  |  |  |  |  |  |  |  |  |  |  |  |
|  |  | Panzihua |  | 1 |  |  |  |  |  |  |  |  |  |  |  |  |  |  |  |  |  |  |  |  |  |  |  | 1 |  |  |  |  |  |  |  |  |  |  |  |  |  |
|  | Yunnan | Dali |  |  | 2 |  | 1 |  |  | 1 |  |  |  |  |  |  |  |  |  |  |  |  |  |  |  |  |  |  |  | 15 | 2 | 2 |  | 1 |  |  |  |  |  |  |  |
|  |  | Fumin |  |  |  |  |  |  |  |  |  |  |  |  |  |  |  |  |  |  |  |  |  |  |  |  |  |  |  |  |  |  |  | 3 |  | 4 | 6 |  | 2 | 1 |  |
|  |  | Kunming |  | 8 | 2 | 4 | 1 |  | 8 | 1 |  | 4 |  |  | 1 |  |  |  |  |  |  |  |  |  | 2 | 2 | 1 |  |  |  |  |  |  | 9 | 3 | 2 |  |  |  |  |  |
|  |  | Shiping |  |  |  |  | 1 |  |  |  |  | 1 |  |  | 1 |  |  |  |  |  |  |  |  |  |  | 2 |  |  |  |  |  |  |  | 9 | 18 |  |  | 1 |  | 2 |  |
| **Japan** | Nagano | Hasuike |  |  |  |  |  |  |  |  |  |  |  |  |  |  |  |  |  |  |  |  |  |  |  |  |  |  |  |  |  |  |  |  |  |  |  |  |  |  | 5 |
|  | Nara | Nara | 3 |  |  |  |  | 2 |  |  |  |  |  |  |  |  | 2 | 1 | 1 |  |  | 1 | 4 |  |  |  |  |  |  |  |  |  |  |  |  |  |  | 1 |  |  |  |
|  | Tokyo | Hachioji | 5 |  |  |  |  | 40 |  |  |  |  | 2 | 5 |  | 3 | 5 | 2 | 2 | 3 | 2 | 1 | 2 | 2 |  |  | 1 | 1 |  |  |  |  |  |  | 1 |  |  |  |  |  |  |
|  |  | **Total** | **8** | **12** | **6** | **12** | **5** | **42** | **8** | **10** |  | **5** | **2** | **5** | **2** | **3** | **7** | **3** | **3** | **3** | **2** | **2** | **6** | **2** | **2** | **4** | **2** | **2** |  | **15** | **2** | **2** |  | **22** | **22** | **6** | **6** | **2** | **2** | **3** | **5** |

**Table S2: GenBank accessions and related information of *COI* and *ITS2* haplotypes described in this study.** Information to *Ganaspis* cf. *brasiliensis* *COI* and (where available) *ITS2* gene sequences of different haplotypes submitted to GenBank (accession numbers). “Extended Group 1” and “Extended Group 3” refer to the molecular clustering observed in this study (see Results and Fig. 1). The haplotype groups A-D refer to variations in variable codons. The parasitoids were originally collected during surveys conducted in 2015 and 2016 at different locations in China and Japan. Sequenced individuals were either those (i) directly collected in the field, (ii) taken from a post-collection laboratory rearing, or (iii) those used in the experiments of this study. The provider of the individuals was either the collector or an intermediate for parasitoids from the laboratory rearing and those used in the experiments. Descriptors for haplotypes and the identification number refer to internal identifiers at the Institute Sophia Antipolis.

| ***COI*** **haplotype** | **Accession number** | ***ITS2* haplotype** | **Accession number** | **Country** | **Province** | **Location** | **Individual background** | **Year** | **Provider** | **Identification number** |
| --- | --- | --- | --- | --- | --- | --- | --- | --- | --- | --- |
| **Extended Group 1** | | | | | | | | | | |
| Amino acid Haplotype A | | | | | | | | | | |
| Hap_042 | MT732399 | its2_a | MT732519 | Japan | Tokyo | Hachioji | Field | 2016 | Borowiec N & Ris N | 28659 |
| Hap_053 | MT732421 | its2_a | MT732533 | China | Sichuan | Dazhou | Field | 2016 | Girod P | 32714 |
| Hap_054 | MT732422 | its2_a | MT732534 | China | Beijing | Jiu | Field | 2016 | Girod P | 32717 |
| Hap_057 | MT732407 | its2_a | MT732523 | China | Yunnan | Kunming | Field | 2015 | Kenis M | 30668 |
| Hap_058 | MT732418 | its2_a | MT732531 | China | Yunnan | Kunming | Field | 2016 | Girod P | 32693 |
| Hap_059 | MT732398 | its2_a | MT732518 | Japan | Tokyo | Hachioji | Field | 2016 | Borowiec N & Ris N | 28653 |
| Hap_089 | MT732424 | its2_a | MT732535 | China | Yunnan | Kunming | Experiment | _ NA | Girod P | 34687 |
| Hap_155 | MT732419 | its2_a | MT732532 | China | Yunnan | Dali | Field | 2016 | Girod P | 32706 |
| Amino acid Haplotype B | | | | | | | | | | |
| Hap_037 | MT732396 | its2_a | MT732517 | China | Yunnan | Shiping | Rearing | NA | Girod P | 26759 |
| Hap_043 | MT732402 | _NA | NA | Japan | Tokyo | Hachioji | Field | 2016 | Borowiec N & Ris N | 28665 |
| Hap 044 | MT732410 | its2 a | MT732526 | Japan | Tokyo | Hachioji | Rearing | NA | Girod P | 31056 |
| Hap_050 | MT732397 | _NA | NA | China | Yunnan | Shiping | Rearing | _ NA | Girod P | 27501 |
| Hap_061 | MT732401 | its2_a | MT732521 | Japan | Tokyo | Hachioji | Field | 2016 | Borowiec N & Ris N | 28664 |
| Hap_064 | MT732400 | its2_a | MT732520 | Japan | Tokyo | Hachioji | Field | 2016 | Borowiec N & Ris N | 28660 |
| Hap_065 | MT732414 | its2_b | MT732528 | Japan | Nara | Nara | Field | 2016 | Borowiec N & Ris N | 31099 |
| Hap_069 | MT732403 | _NA | NA | Japan | Tokyo | Hachioji | Field | 2016 | Borowiec N & Ris N | 28667 |
| Hap_112 | MT732411 | its2_a | MT732527 | Japan | Tokyo | Hachioji | Field | 2016 | Borowiec N & Ris N | 31077 |
| Hap_113 | MT732412 | _NA | NA | Japan | Tokyo | Hachioji | Field | 2016 | Borowiec N & Ris N | 31079 |
| Hap_117 | MT732413 | _NA | NA | Japan | Tokyo | Hachioji | Field | 2016 | Borowiec N & Ris N | 31084 |
| Hap_132 | MT732415 | its2_a | MT732529 | Japan | Tokyo | Hachioji | Field | 2016 | Borowiec N & Ris N | 31120 |
| Hap_133 | MT732404 | _NA | NA | Japan | Tokyo | Hachioji | Rearing | _ NA | Borowiec N | 28725 |
| Hap_144 | MT732416 | its2_a | MT732530 | China | Yunnan | Kunming | Field | 2015 | Girod P | 31710 |
| Hap_150 | MT732417 | _NA | NA | China | Yunnan | Shiping | Field | 2015 | Girod P | 31719 |
| Hap_156 | MT732420 | _NA | NA | China | Yunnan | Kunming | Field | 2016 | Girod P | 32707 |
| Hap_165 | MT732423 | _NA | NA | China | Sichuan | Panzihua | Field | 2016 | Girod P | 32728 |
| Amino acid Haplotype C | | | | | | | | | | |
| Hap_078 | MT732406 | its2_a | MT732522 | China | Yunnan | Dali | Field | 2016 | Kenis M | 30631 |
| Hap_169 | MT732425 | _NA | NA | China | Yunnan | Dali | Experiment | _ NA | Girod P | 36049 |
| Hap_177 | MT732428 | _NA | NA | China | Yunnan | Dali | Experiment | _ NA | Girod P | 37048 |
| **Extended Group 3** | | | | | | | | | | |
| Amino acid Haplotype D | | | | | | | | | | |
| Hap_040 | MT732408 | its2_c | MT732524 | China | Yunnan | Kunming | Field | 2015 | Kenis M | 30671 |
| Hap_080 | MT732409 | its2_c | MT732525 | China | Yunnan | Kunming | Field | 2015 | Kenis M | 30675 |
| Hap_142 | MT732427 | its2_c | MT732537 | China | Yunnan | Fumin | Experiment | NA | Girod P | 36063 |
| Hap_170 | MT732426 | its2_c | MT732536 | China | Yunnan | Fumin | Experiment | NA | Girod P | 36061 |
| Hap_174 | MT732405 | _NA | NA | Japan | Nara | Nara | Rearing | NA | Girod P | 28786 |
| Hap_179 | MT732429 | _NA | NA | China | Yunnan | Fumin | Experiment | NA | Girod P | 37065 |
| Hap_180 | MT732430 | its2_c | MT732538 | China | Yunnan | Fumin | Experiment | NA | Girod P | 37068 |
| Hap_181 | MT732431 | its2_c | MT732539 | Japan | Nagano | Hasuike | Experiment | NA | Girod P | 37074 |

**Table S3: Statistical results of the no-choice bioassay testing specificity of *Ganaspis* cf. *brasiliensis* from different origins and genetic groups.** Results of the logistic regression analyzing the influence of the independent variables parasitoid origin (seven locations in Asia representing two genetic groups), three oviposition substrates (*Drosophila suzukii* feeding on either blueberries or artificial diet and *D. melanogaster* feeding on artificial diet), and their interaction on apparent parasitism by *G*. cf. *brasiliensis* during a non-choice bioassay.

| **Independent variable** | **χ^2^-value** | **df** | ***P*-value** |
| --- | --- | --- | --- |
| Origin | 175.13 | 6, 396 | <0.0001 |
| Oviposition substrate | 8.41 | 2, 396 | 0.0149 |
| Origin × Substrate | 106.04 | 12, 396 | <0.0001 |

**Table S4:** **Statistical results of the no-choice bioassay testing the ability of *Ganaspis* cf. *brasiliensis* from different genetic groups to parasitize non-target species.** Results of the logistic regression analyzing the influence of the independent variables parasitoid origin and genetic background (three locations in Asia representing two genetic groups), non-target host species (*Drosophila melanogaster* and *D. simulans*), oviposition substrate (blueberries or artificial diet), and all possible interactions on apparent parasitism by *G*. cf. *brasiliensis* during a non-choice bioassay.

| **Independent variable** | **χ^2^-value** | **df** | ***P*-value** |
| --- | --- | --- | --- |
| Origin | 124.39 | 2, 105 | <0.0001 |
| Host species | 1.50 | 1, 105 | 0.2208 |
| Oviposition substrate | 1.65 | 1, 105 | 0.1995 |
| Origin × Species | 13.39 | 2, 105 | 0.0012 |
| Origin × Substrate | 13.86 | 2, 105 | 0.0010 |
| Species × Substrate | 0.03 | 1, 105 | 0.8638 |
| Origin × Species × Substrate | 2.78 | 2, 105 | 0.2489 |

**Table S5: Statistical results of the three-choice bioassay testing specificity of *Ganaspis* cf. *brasiliensis* from different genetic groups.** Results of logistic regressions (one per parasitoid origin) analyzing the influence of three oviposition substrates (*Drosophila suzukii* or *D. melanogaster* larvae feeding on blueberry, and *D. melanogaster* larvae feeding on artificial diet) as independent variable on apparent parasitism by *G.* cf. *brasiliensis* from three locations in Asia (Tokyo, Xining, and Hasuike) representing two genetic groups (extended G1 and merged G3-4; see Results and Fig. 1) during a three-choice bioassay.

| **Parasitoid origin** | **Number of**  **responding**  **females** | **χ^2^-value** | **df** | ***P*-value** |
| --- | --- | --- | --- | --- |
| Tokyo (G1) | 11 | 24.43 | 2, 27 | <0.0001 |
| Xining (G1) | 14 | >1000 | 2, 36 | <0.0001 |
| Hasuike (G3-4) | 14 | 2.10 | 2, 37 | 0.3501 |

**Table S6: Statistical results of the four-choice bioassay testing specificity of *Ganaspis* cf. *brasiliensis* from different genetic groups.** Results of two logistic regressions analyzing the influence of the independent variables host species (*Drosophila suzukii* or *D. melanogaster*), fruit status (fresh or decomposing), and their interaction on apparent parasitism by *G.* cf. *brasiliensis* from two locations in Asia, Tokyo (n=16) and Hasuike (n=21), representing two genetic groups (extended G1 and merged G3-4; see Results and Fig. 1) during a four-choice bioassay.

| **Independent variable** | **χ^2^-value** | **df** | ***P*-value** |
| --- | --- | --- | --- |
| **Tokyo (G1)** | | | |
| Host species | 2.79 | 1, 58 | 0.0950 |
| Fruit status | 7.52 | 1, 58 | 0.0061 |
| Host × Status | 0.69 | 1, 58 | 0.4048 |
| **Hasuike (G3-4)** | | | |
| Host species | 7.70 | 1, 78 | 0.0055 |
| Fruit status | 21.21 | 1, 78 | <0.0001 |
| Host × Status | 4.63 | 1, 78 | 0.0314 |

**Table S7:** Mean genetic divergences based on the Kimura 2 parameters distance within and between genetic clusters described in this study. Within the merged group 3-4, putative pseudogenes correspond to the haplotype carrying non-synonymous mutations in the amino-acid sequence (cf. asterisks in Figure 1). G2 and G4 (sensu stricto) were represented by only one haplotype (respectively LC122441.1 and LC122447.1 in Figure 1), preventing the calculation of the within-cluster divergences.

| **Genetic cluster** | **Extended Group 1** | **Merged Group 3-4** | **Group 2** | **Group 4** | **Group 5** | **Putative pseudogenes of merged Group 3-4** |
| --- | --- | --- | --- | --- | --- | --- |
| **Extended Group 1** | 0.0104 | 0.0556 | 0.0538 | 0.0569 | 0.0491 | 0.0546 |
| **Merged Group 3-4** | 0.0556 | 0.0076 | 0.0375 | 0.0174 | 0.0206 | 0.0150 |
| **Group 2** | 0.0538 | 0.0375 | - | 0.0390 | 0.0337 | 0.0404 |
| **Group 4** | 0.0569 | 0.0174 | 0.0390 | - | 0.0218 | 0.0211 |
| **Group 5** | 0.0491 | 0.0206 | 0.0337 | 0.0218 | 0.0013 | 0.0238 |
| **Putative pseudogenes of merged Group 3-4** | 0.0546 | 0.0150 | 0.0404 | 0.0211 | 0.0238 | 0.0151 |


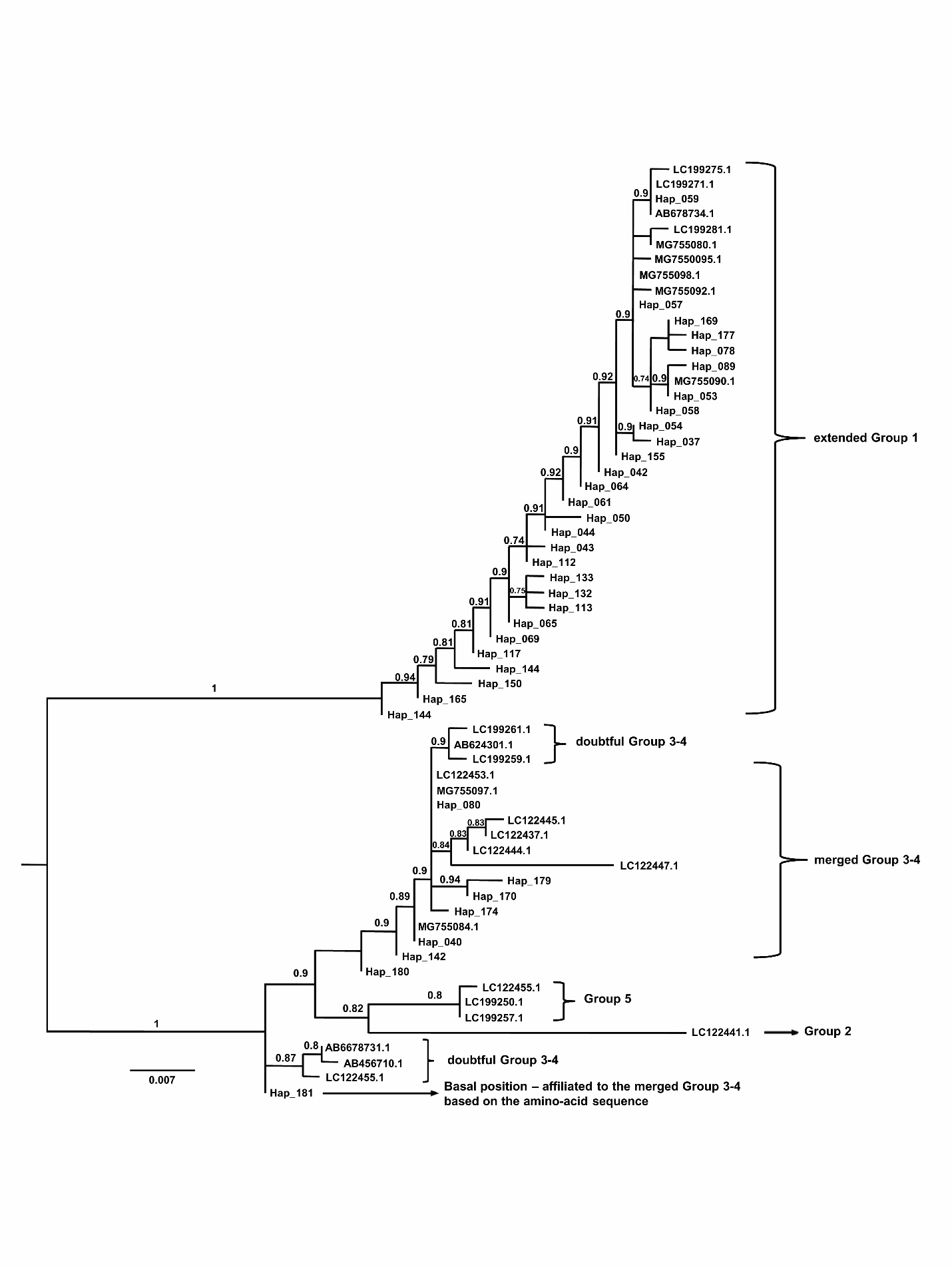


**Fig. S1: Maximum Likelihood tree for *COI* of *Ganaspis* cf. *brasiliensis***. The molecular groups refer to the molecular clustering observed in this study (see Results and Fig. 1). “Doubtful Group 3-4” refers to sequences for which a change in the amino acid sequence was observed.


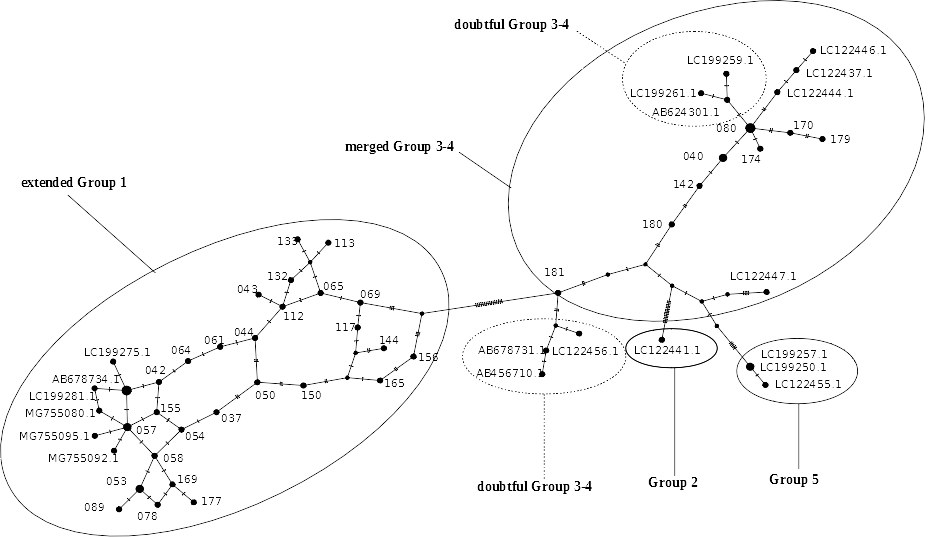


**Figure S2: Haplotype network established from *COI* sequences of *Ganaspis* cf. *brasiliensis***. Haplotype network based on the Median Joining method with Ꜫ=0. The molecular groups refer to the molecular clustering observed in this study (see Results and Fig. 1). “Doubtful Group 3-4” refers to sequences for which a change in the amino acid sequence was observed.
